# Slingshot feeding of the goblin shark *Mitsukurina owstoni* (Pisces: Lamniformes: Mitsukurinidae)

**DOI:** 10.1038/srep27786

**Published:** 2016-06-10

**Authors:** Kazuhiro Nakaya, Taketeru Tomita, Kenta Suda, Keiichi Sato, Keisuke Ogimoto, Anthony Chappell, Toshihiko Sato, Katsuhiko Takano, Yoshio Yuki

**Affiliations:** 1 Hokkaido University, 3-1-1, Minato-cho, Hakodate, Hokkaido 041-8611, Japan; 2 Okinawa Churashima Research Center, 888 Ishikawa, Motobu-cho, Okinawa 905-0206, Japan; 3 Kesen-numa Shark Museum, 7-13, Uoichiba-mae, Kesen-numa, Miyagi 998-0037, Japan; 4 Hokkaido University Museum, 3-1-1, Minato-cho, Hakodate, Hokkaido 041-8611, Japan; 5 Marine Business Division, Okabe Co., Ltd., Oki-gun, Shimane 684-0404, Japan; 6 Shimonoseki Marine Science Museum, 6-1 Arcaport, Shimonoseki, Yamaguchi 750-0036, Japan; 7 2 Place de la republique, 60660 cramoisy, France; 8 NHK (Japan Broadcasting Association), Naha, Okinawa, Japan; 9 NHK (Japan Broadcasting Association), Tokyo, Japan

## Abstract

Five striking and prey capture events of two goblin sharks were videotaped at sea for the first time, showing their extraordinary biting process. The goblin sharks swung their lower jaw downward and backward to attain a huge gape and then rapidly protruded the jaws forward a considerable distance. The jaws were projected at a maximum velocity of 3.1 m/s to 8.6–9.4% of the total length of the shark, which is by far the fastest and greatest jaw protrusion among sharks. While the jaws were being retracted, the mouth opened and closed again, which was considered a novel feeding event for sharks. Phylogenetic evidence suggested that their feeding behavior has evolved as an adaptation to food-poor deep-sea environments, possibly as a trade-off for the loss of strong swimming ability.

The goblin shark *Mitsukurina owstoni* is a deep-sea shark that was originally described based on a 1070 mm specimen, collected from Japan^1^. The original drawing of the species was made with its mouth maximally protruded, which gave an unusual appearance to the shark that inspired the name “goblin.”

Because of its extraordinary appearance, the goblin shark has always been of keen interest, especially regarding its feeding behavior. The goblin shark is believed to catch its prey by projecting its very protrusible jaws^2^,^3^,^4^, but its actual feeding behavior has never been observed.

The feeding behaviors of sharks are mostly based on inferences from anatomical studies of specimens^5^,^6^,^7^,^8^,^9^,^10^ and/or experimental studies of live specimens in a few representative species^9^,^11^,^12^,^13^,^14^. The feeding behavior of the goblin shark has been inferred from drawings^1^, photographic evidence^15^, and anatomical aspects^8^ of specimens. Because of the inherent difficulties of *in situ* studies on deep-sea sharks, their actual feeding behaviors are poorly understood^16^,^17^.

The goblin shark is one of the least known sharks, because of the difficulty in accessing its deep water habitat and the resulting infrequency of encounters with humans^4^,^18^. In 2008 and 2011, diving teams from NHK (Japan Broadcasting Association) were successful in recording five striking and predatory behaviors of two goblin sharks at sea, the first such observation of jaw kinetics since the discovery of the species in the 19^th^ century. Here we describe *in situ* striking and predatory events of the goblin shark, and analyze the details of the events during the motion. We then discuss the selective pressures that perhaps adapted the feeding tactics of this shark to the deep-sea environment.

## Results

**Figure 1:**
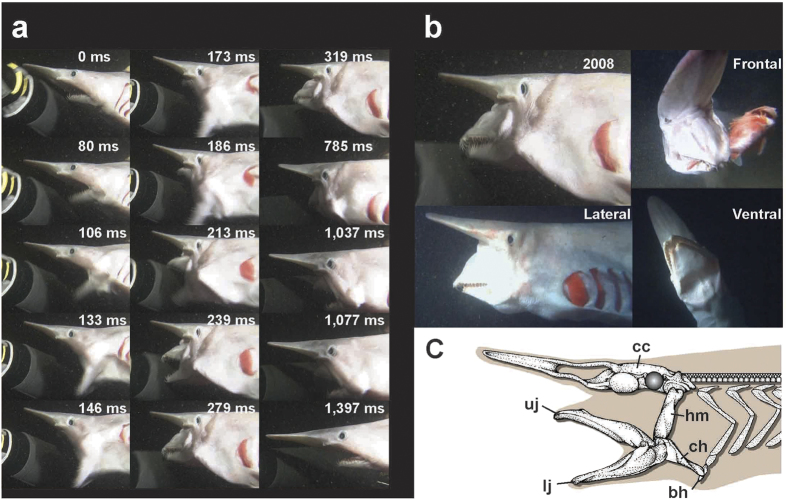
Striking and prey capture behaviors of the goblin shark. **(a)** Striking behavior of the 2008 shark arranged in sequential order in milliseconds (ms). Resting phase, 0 ms; expansive phase, 0–146 ms; compressive phase, 146–785 ms (shooting stage, 146–239 ms; grasping stage, 239–319 ms; holding stage, 319–785 ms); recovery phase, 785–1,397 ms (re-opening stage, 785–1,077 ms; re-closing stage, 1,077–1,397 ms). **(b)** Lateral view of peak protrusion in the 2008 shark (279 ms in Fig. 1a), and lateral, frontal, and ventral views of peak protrusions in the 2011 shark. “Lateral” and “frontal” views are prey capture behaviors on a bait fish. **(c)** Skeletal elements related to predatory behavior of the goblin shark. bh, bathyhyal cartilage; cc, chondrocranium; ch, ceratohyal cartilage; hm, hyomandibular cartilage; lj, lower jaw (Meckel’s) cartilage; uj, upper jaw (palatoquadrate) cartilage. (**a**,**b**) Photographs are used with permission of NHK, NEP and the Discovery Channel).

Striking events of the 2008 shark (Fig. 1a) are described below as a standard measure, with additional and supplemental striking and predatory events taken from the 2011 shark (Fig. 1b). The skeletal structure and terminology of the cartilages related to feeding are given in Fig. 1c.

### Resting phase (Figs 1–4; 0 ms)

**Figure 2:**
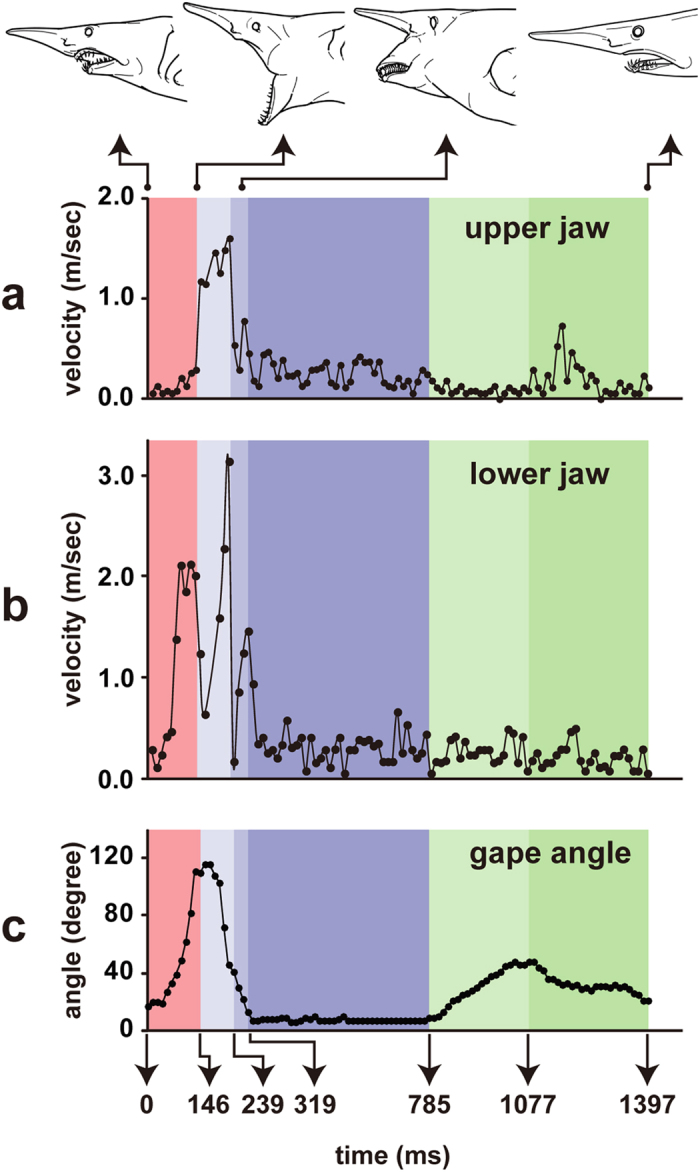
Striking velocities of upper jaw (**a**) and lower jaw (**b**), and gape angles (**c**) in one biting sequence of the 2008 shark.

**Figure 3:**
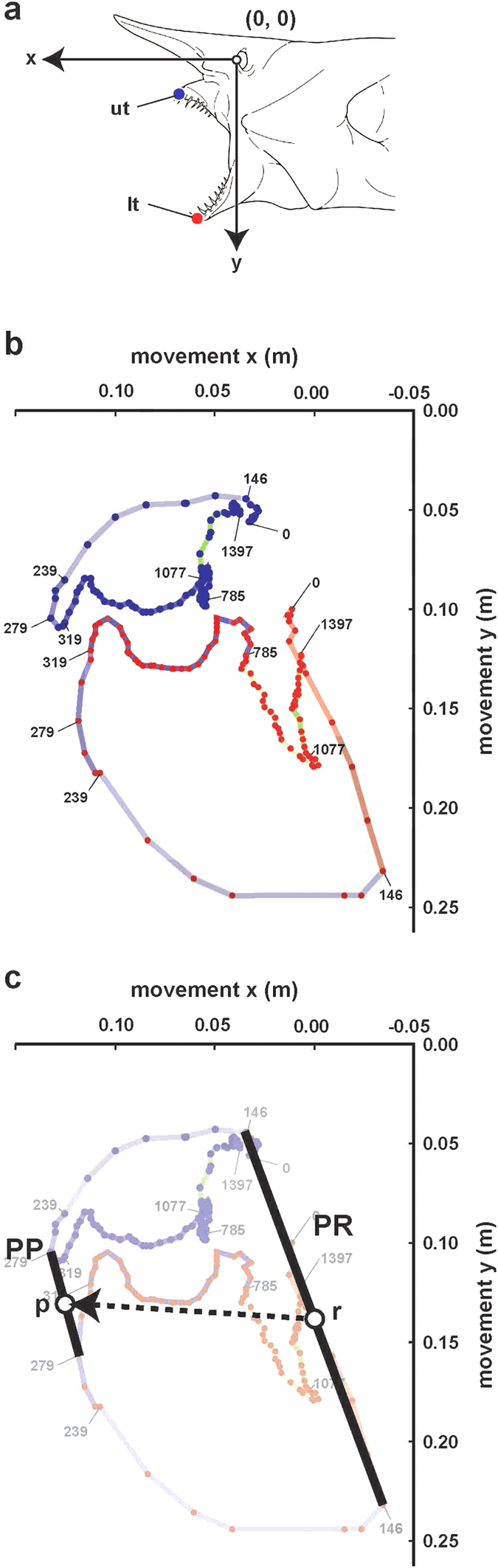
Kinematic tracks of jaws in the 2008 shark. **(a)** Diagram showing origin (0, 0), and anterior tips of upper (ut) and lower (lt) jaws. **(b)** Kinematic tracks of anterior tips of upper jaw (upper loop) and lower jaw (lower loop) in milliseconds. Resting phase, 0 ms; expansive phase, 0–146 ms; compressive phase, 146–785 ms (shooting stage, 146–239 ms; grasping stage, 239–319 ms; holding stage, 319–785 ms); recovery phase, 785–1,397 ms (re-opening stage, 785–1,077 ms; re-closing stage, 1,077–1,397 ms); peak retraction, 146 ms; peak protrusion, 279 ms. (**c**) Direction of jaw protrusion (broken arrow). PP, gape line at peak protrusion; PR, gape line at peak retraction; p, midpoint of PP; r, midpoint of PR.

**Figure 4:**
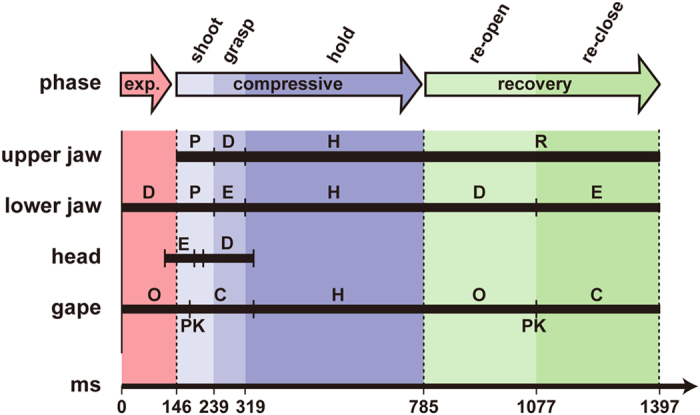
Composite diagram of striking events and time in the 2008 shark. C, closing; D, depression; E, elevation; H, holding; O, opening; P, protrusion; PK, peak; R, retraction. Sequence is shown as a black bar.

The jaws were slightly open (Fig. 1a), with a gape angle of ca. 20° (Fig. 2c). The head was kept straight relative to the body axis (Figs 1, 2, 3, 4).

### Expansive phase (Figs 1–4; 0–146 ms)

#### Jaw movements

The jaws quickly opened (Figs 1a, 2c and 3b). The anterior end of the upper jaw cartilage (palatoquadrate cartilage, Fig. 1c) remained in the resting position during the expansive phase (Figs 2a and 3b), but its posterior end was strongly depressed from 106 ms after the onset of the striking behavior. The lower jaw was depressed at a velocity less than 0.50 m/s until 80 ms, and then swung posteroventrally at about 2.0 m/s (Fig. 2b), attaining peak retraction at 146 ms (Figs 1a and 3b). The upper and lower jaw cartilages were rotated outward to expose the teeth (also observable in the ventral view of the 2011 shark).

#### Gape angle

The gape angle (Fig. 2c) increased from ca. 20° to 27° during the first third of the expansive phase (0–53 ms), and then increased to 111° at 133 ms.

#### Head movements

The head (Fig. 1a) was elevated from 106 ms.

#### Other events

The eye transformed from round to vertically elliptical from 106 ms. The gill openings opened at 133 ms, with gill filaments visible. The hyomandibular cartilage (Fig. 1c) remained in the resting position during 0–106 ms, and its distal end was rotated anteroventrally, beginning at 106 ms. The basihyal cartilage (Fig. 1c), which supports the tongue, and the distal end of ceratohyal cartilage (Fig. 1c), which links the basihyal and jaw cartilages, were pulled posteroventrally (also observable in the ventral view of the 2011 shark). The basihyal cartilage pushed the intermandibular plates ventrally during 0–27 ms, posteroventrally during 27–120 ms, and posteriorly during 120–146 ms, resulting in the depression (0–27 ms) and posteroventral rotation (27–146 ms) of the lower jaw (also observable in the ventral and oblique views of the 2011 shark). The right and left intermandibular plates were in contact until the end of the expansive phase (also observable in the ventral view of the 2011 shark).

### Compressive phase (Figs 1–4; 146–785 ms)

The compressive phase was subdivided herein into the shooting, grasping, and holding stages. The shooting stage is the first stage of the compressive phase, defined as the duration that the jaws are protruded anteriorly, or the horizontal moments of anterior ends of jaws exceed vertical moments. The grasping stage is the second stage of the compressive phase, defined as the duration that the jaws are closed, or the vertical moments of the anterior ends of jaws exceeding the horizontal moments. The holding stage is the last stage of the compressive phase, defined as the duration that the jaws are kept closed after the grasping stage.

#### Shooting stage (146–239 ms)

##### Jaw movements

The jaws (Figs 1a and 3b) were extensively and rapidly protruded. The upper jaw was projected forward, attaining a maximum velocity of 1.60 m/s at 239 ms (Fig. 2a). The lower jaw was swung anterodorsally from the peak retraction and protruded quickly forward at a maximum velocity of 3.14 m/s at 239 ms (Fig. 2b).

##### Gape angle

The gape angle (Fig. 2c) slightly increased to 116° during 160–173 ms, attaining the maximum gape, and abruptly decreased to 41° at 239 ms.

##### Head movements

The head (Fig. 1a) was elevated 12° from the resting position at 186 ms (13° in the lateral view of the 2011 footage) and was depressed from 213 ms to 239 ms (continuously to 372 ms).

##### Other events

The eye transformed from round to vertically elliptical (maximally at 226–239 ms), but the eyeball did not rotate. The skin between the eye and 1st gill opening was strongly pulled anteriorly by the rapid protrusion of the jaws, and the first gill opening transformed to crescent shape, exposing the gill filaments (maximally at 239 ms). The basihyal cartilage was separate from the intermandibular plates (also observable in the ventral, lateral, and oblique views of the 2011 shark). The distal end of the hyomandibular cartilage was swung anteroventrally, while the proximal end of the ceratohyal cartilage swung anteriorly (also observable in the lateral and oblique views of the 2011 shark). The basihyal cartilage and the distal end of the ceratohyal cartilage remained in the same position (also in the ventral, frontal, lateral, and oblique views of the 2011 shark). The right and left intermandibular plates were completely separate from each other (also in the ventral view of the 2011 shark).

#### Grasping stage (239–319 ms)

##### Jaw movements

The upper and lower jaws (Figs 1a and 3b) were maximally extended and closed below the elevated snout. The upper jaw was depressed at a velocity of 0.1–0.8 m/s (Fig. 2a), and the lower jaw was elevated at a velocity of 0.8–1.5 m/s (Fig. 2b). The jaws reached their peak protrusion at 279 ms. The longitudinal travel distances of the upper jaw tip to the point of peak protrusion (279 ms) were 111 mm from the resting position (0 ms) and 114 mm (33.2% of head length) from the onset of the shooting stage (146 ms), and those of the lower jaw were 121 mm (35.5% of head length) and 171 mm (49.9% of head length), respectively. The mouth was closed under the elevated snout at 319 ms.

##### Gape angle

The gape angle (Fig. 2c) decreased from 41° at 239 ms to 7–8° at 319 ms.

##### Head movements

The head (Fig. 1a) was depressed during the grasping stage.

##### Other events

The eye was vertically elliptical. The skin between the eye and 1^st^ gill opening was strongly pulled forward. The hyomandibular, ceratohyal, and basihyal cartilages remained in same positions as those at the end of shooting stage (also observable in the lateral and oblique views of the 2011 shark). The right and left intermandibular plates were widely separate from each other (also in the ventral view of the 2011 shark). The palatonasal ligament was maximally stretched at 279 ms (also in the lateral view of the 2011 shark).

#### Holding stage (319–785 ms)

##### Jaw movements

The jaws (Figs 1a and 3b) were kept closed below the elevated snout, and gradually retracted.

##### Gape angles

The gape angle (Fig. 2c) was maintained at 6–10° throughout the holding stage.

##### Head movements

The head (Fig. 1a) was depressed until 399 ms, after which it returned to the original position.

##### Other events

The eye was vertical and elliptical. The skin between the eye and 1^st^ gill opening was kept strongly pulled forward. The hyomandibular, ceratohyal, and basihyal cartilages stayed in the same position (also observable in the ventral, lateral, and oblique views of the 2011 shark). The right and left intermandibular plates were widely separate from each other (also in the ventral view of the 2011 shark).

### Recovery phase (Figs 1–4; 785–1,397 ms)

The recovery phase was subdivided into the re-opening and re-closing stages. The re-opening stage is the first half of the recovery phase, defined as the duration from the onset of mouth retraction to the maximum gape of the jaws in the recovery phase. The re-closing stage is the second half of the recovery phase, defined as the duration from the maximum gape in the recovery phase to the resting position.

#### Re-opening stage (785–1,077 ms)

##### Jaw movements

The mouth (Figs 1a and 3b) opened, with the upper jaw slowly elevated, and the lower jaw extensively depressed. The jaws were gradually retracted.

##### Gape angle

The gape angles (Fig. 2c) increased from 10° at the onset of re-opening to 46–48° at the end of the re-opening (1,011–1,077 ms).

##### Head movements

No noticeable movements were observed.

##### Other events

The skin between the eye and 1^st^ gill opening returned to its original condition. The distal part of the hyomandibular cartilage and the proximal part of the ceratohyal cartilage were swung posterodorsally as the jaws were retracted (also observable in the lateral view of the 2011 shark). The right and left intermandibular plates were widely separate and gradually retracted until the basihyal cartilage was embraced by the intermandibular plate (also in the oblique view of the 2011 shark).

#### Re-closing stage (1,077–1,397 ms)

##### Jaw movements

The mouth (Figs 1a and 3b) was gradually closed, and the jaws returned to the resting position, with the upper jaw slowly elevated and the lower jaw elevated.

##### Gape angle

The gape angle (Fig. 2c) decreased from 46–48° to ca 20° at the resting position (1,397 ms).

##### Head movements

No noticeable movements were observed.

##### Other events

The eye, and the skin between the eye and 1^st^ gill opening returned to their original condition. The distal part of the hyomandibular cartilage was elevated (also observable in the lateral and oblique views of the 2011 shark), and the proximal part of the ceratohyal cartilage was pulled posteriorly to the resting position (also in the oblique view of the 2011 shark). The right and left intermandibular plates approached and finally contacted each other medially. The basihyal cartilage was completely encompassesd by the intermandibular plate (also in the oblique view of the 2011 shark).

## Discussion

The goblin shark belongs to the order Lamniformes (mackerel sharks). The mackerel sharks are generally predatory sharks that capture prey animals by suction, ramming, or a combination of these^11^, followed by or together with biting. Our video footage indicates that the goblin shark is a ram feeder, because the jaws were protruded after the full opening of the gape, and because the gill openings were open throughout the biting, indicating that the pressure in the oral and pharyngeal cavities was always positive (Fig. 1a).

The kinematic tracks of the goblin shark (Fig. 3) revealed the movements and relative positions of the upper and lower jaws during a strike. The lower jaw has more operative and complex movements than those of the upper jaw, and most likely plays an important role in capturing and manipulating the prey animals. The direct distances traveled by the anterior tips of the upper and lower jaws from the resting position (0 ms in Figs 1a and 3b) to the peak protrusion (279 ms in Figs 1a, 3b) were 8.6% of total length (TL) (or 32.3% of head length [HL]) for the upper jaw and 9.4% TL (35.5% HL) for the lower jaw. The known upper jaw protrusions for other sharks are 1.6% TL (converted based on the HL/TL ratio of the species^3^; 9% HL) in the broadnose sevengill shark *Notorynchus cepedianus*^11^, 1.4% TL^3^ (7% HL) in the horn shark *Heterodontus francisci*^11^, 2.0% TL^3^ (7.8% HL) in the blacktip shark *Carcharhinus limbatus*^11^, 4.0% TL^3^ (18% HL) in the lemon shark *Negaprion brevirostris*^11^, 2.2% TL^3^ (10% HL) in the bonnethead shark *Sphyrna tiburo*^11^, and 3.8% TL (29–30% HL) in the spiny dogfish *Squalus acanthias*^14^,^19^. The jaw protrusions of three carpetsharks were reported to be 1.6% TL (converted based on CL/TL ratio of the species^20^; 12% of the chondrocranial length (CL)) for the nurse shark *Ginglymostoma cirratum*^7^, 0.9% TL^20^ (9% CL) for the epaulette shark *Hemiscyllium ocellatum*^7^, and ca. 4.0% TL^20^ (32.6% CL) for the spotted wobbegong *Orectolobus maculatus*. These facts clearly indicate that the jaws of the goblin shark are extremely protrusile, being 2.1–9.5 times greater than those of the other sharks.

The time to maximum gape is generally shorter in the suction-feeding sharks (30–64 ms) than in the ram-feeding sharks (81–162 ms)^16^, and the present goblin shark (160–173 ms) falls almost within the range of the ram-feeding sharks. Immediately after the lower jaw reached the peak retraction (146 ms), both jaws were rapidly protruded, i.e., the upper jaw at a maximum speed of 1.60 m/s (Fig. 2a), and the lower jaw at 3.14 m/s (Fig. 2b). The velocity of the lower jaw was 1.6–2 times greater than that of the upper jaw because the lower jaw accompanies the upward swinging motion. The strike velocities of the lesser electric ray *Narcine brasiliensis*, which employs rapid suction feeding by the extreme jaw protrusion, were only 0.28 m/s for the lower jaw and 0.87 m/s for the upper jaw^21^. The sling-jaw wrasse *Epibulus insidiator* is a well-known example among fishes in having rapid and extensive jaw protrusion for capturing the prey, and has its maximum protrusion velocity of 2.31 m/s^22^. These facts demonstrate that the goblin shark projects its jaws toward the prey at an extremely high speed (maximally 3.14 m/s).

The gape angles changed greatly during the strike (Fig. 2c). Curiously, the mouth was opened again (46–48°) from the onset of the recovery phase (785 ms) to the end of the re-opening stage (1,077 ms), and gradually closed to return to the resting phase (20°, 1,397 ms). The re-opening and re-closing actions were also consistently observed in the 2011 shark. These actions have never been observed in other shark species, suggesting that this is a novel event in shark feeding behavior. The functional importance of the re-opening and re-closing actions in the goblin shark might be related to the extreme jaw protrusion and the relative movements of the basihyal cartilage and intermandibular plate.

Figure 3c shows the gape line (a line connecting the anterior tips of the upper and lower jaws) at peak retraction (PR) and peak protrusion (PP), and its movements. The line moves forward from PR during the shooting stage to reach PP, and the broken arrow indicates the movements of the midpoint of the gape line from PR to PP. This figure indicates that the jaws, or the gape of the goblin shark, are protruded forward and even slightly upward, and that the mouth is closed to the swimming direction (Fig. 1b). The other ram feeding sharks project the jaws ventrally or anteroventrally, as observed in the great white shark *Carcharodon carcharias*^23^, lemon shark *Negaprion brevirostris*^11^,^24^, sandtiger shark *Carcharias taurus*^16^, blacktip shark *Carcharhinus limbatus*^17^, spiny dogfish *Squalus acanthias*^11^,^14^,^19^, cookiecutter shark *Isistius brasiliensis*^5^, and viper dogfish *Trigonognathus kabeyai*^6^. Therefore, the goblin shark is also unique in the direction of its jaw protrusion.

**Figure 5:**
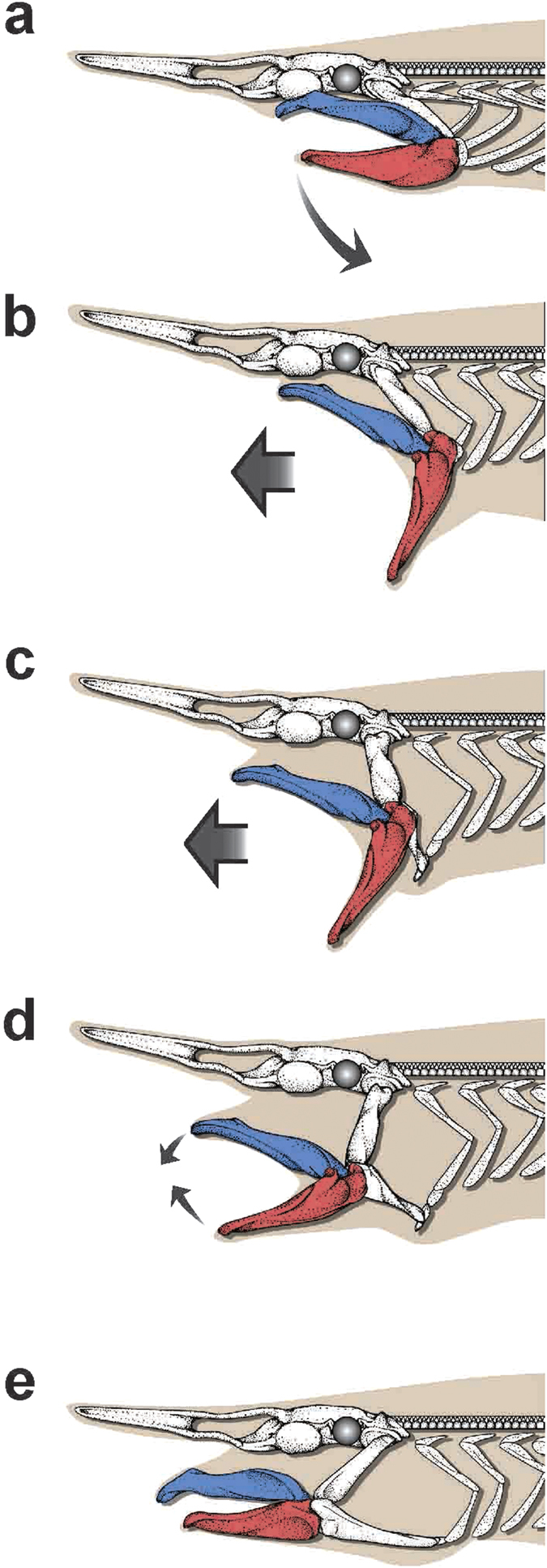
Slingshot feeding of the goblin shark. **(a)** Resting phase; **(b)** peak retraction; **(c)** shooting stage; **(d)** onset of grasping stage; **(e)** peak protrusion. Thick arrows indicate movements of jaws, and thin arrows indicate movements of upper and lower jaw cartilages.

The present *in situ* video footages reveal the extraordinary novel “slingshot-like” predatory events of the goblin shark. The goblin shark projects its jaws suddenly and momentarily forward for a considerable distance at great velocity (Fig. 5).

Mackerel sharks are generally swift swimmers, with a conical head, a torpedo-shaped body, well-developed body musculature, rigid fins, and a strong lunate caudal fin. They ram-feed on prey animals by increasing their swimming speed using a lunate caudal fin, overtaking the prey until close enough to bite them^24^,^25^. The goblin shark also ram-feeds on its prey, but it is morphologically quite different from the other typical mackerel sharks in having a long flattened head, a slender body, flabby body musculature, small and soft fins, and a weak ribbon-like caudal fin, suggesting a different mode of life from them. The goblin shark is also considered a deep-water inactive and almost neutrally buoyant shark^2^,^3^,^4^,^18^, and it actually swims slowly by undulating the tail region and long caudal fin (*in situ* personal observations).

The goblin shark primarily preys on bony fishes^26^, and two large grenadiers were found in the stomach of a goblin shark examined (YCM-P 12237). The present 2011 shark also snapped a baited rockfish by protruding the jaws (Fig. 1b, lateral and frontal views). The biological roles of the jaw protrusion in sharks include enhanced biting and manipulation of the prey, improved grasping, more effective cutting and gouging, rapid closure of the jaws, and creating suction currents^16^. The rapid and extensive jaw protrusion of the goblin shark may compensate for its apparent lack of ability for fast and sustained swimming to pursue prey. The jaw protrusion of the goblin shark will serve the species to expand the accessible distance to the prey, and enable it to capture the faster swimming prey, allowing it to seize elusive prey. Its very recurved teeth would also prevent the prey escape once seized.

The goblin shark is the only deep-sea shark in the order Lamniformes, inhabiting depths of 270 m to at least 1300 m of the mid-water^4^, or near the continental slopes and seamounts^3^,^18^,^27^, whereas the other mackerel shark families generally live in the insular waters or the epipelagic zone of oceanic waters^2^,^4^,^18^,^27^. The phylogenetic relationships^28^,^29^,^30^,^31^,^32^, depth distribution of the related sharks^2^,^3^,^4^,^18^,^27^ and paleontological facts^33^,^34^,^35^,^36^ strongly suggest that the mackerel sharks have a shallow water origin, and that the goblin shark evolved from a shallow water lamniform ancestor and adapted to the deep-sea environments. The goblin shark is further considered to have developed its slingshot feeding to adapt to the food-poor deep-sea environments and to compensate for the loss of the strong swimming ability that is generally observed in deep-sea fishes^37^.

## Materials and Methods

### Video recordings

Two individuals, both ca. 1300 mm in total length (TL), captured from the Tokyo Underwater Canyon in the Tokyo Bay by the gill nets in 2008 and 2011 each, were used for the behavioral observations at sea. The 2008 shark was captured at depths of 150–350 m, and transported to the Kanaya Fishing Port nearby (6 m in depth, 9 °C), where the shark was released and its striking behavior was videotaped. The detailed recording conditions of the 2011 shark were unavailable, but were almost the same as those of the 2008 shark. Five video recordings of the striking and prey capture events were obtained. The first recording (2008 shark) was taken on January 30, 2008 at a speed of 75.5 fps (frames/secondd). The second to fifth recordings (2011 shark) were recorded on January 24, 2011 at a speed of 300 fps. A Sony HDW750 underwater video camera and an ultra-slow motion camera (Hi-Motion 300 p) from Nac Image Technology Inc. were used in 2008 and 2011, respectively. The first recording was a strike on a diver’s arm, and as it covers the entire striking event, it was used for the detailed descriptions and analyses of the process. The second to fifth recordings include two predatory (pr) and two striking (st) events videotaped from four different angles, i.e., the frontal (pr), lateral (pr), oblique (st), and ventral (st) aspects. As the 2011 recordings only covered part of the events, they were used to reconfirm and supplement the description of the 2008 shark, and to find other events that were not observable in the 2008 footage. The two videotaped individuals above were released after the recordings.

### Definition of prey capture sequence

The prey capture sequence of sharks is divided into following four phases: resting (preparatory), expansive, compressive, and recovery phases^16^,^17^. However, as different events were recognized in the compressive and recovery phases of the goblin shark, the compressive phase was herein subdivided into the shooting, grasping, and holding stages, and the recovery phase was subdivided into the re-opening and re-closing stages.

### Prey capture behavior analysis

The striking events of the 2008 shark were described and analyzed based on the resolved photographic images secured every 13.3 ms. The velocities of the upper and lower jaws were calculated from the video footage of the 2008 shark in the following three steps. 1) Still images were captured from the video images at 75.5 fps using the movie-editing software KMplayer 2.9.4.1.1435 (Jelsoft Enterprises Ltd) (Fig. 1a). 2) The coordinates were measured from each still image using the coordinate-measuring tool in ImageJ (US National Institutes of Health, Bethesda, MD). Coordinates were acquired for the anterior tips of the upper and lower jaws. The horizontal axis (x-axis) was set parallel to the bottom of the frame, and the perpendicular axis (y-axis), vertical to the bottom of the frame. The position of the origin (x = 0, y = 0) was located on the anterior-most point of the eye. The distance between the anterior tip of the snout and the anterior-most point of the eye was scaled at 172 mm, which was estimated from a similar-sized specimen (HUMZ 204615, 1315 mm TL). 3) Moving velocities (v_x_, v_y_) [m/s, m/s] of the jaw tips were calculated for each point on the upper and lower jaw as (v_x_, v_y_) = (∆x × 75.5, ∆y × 75.5), where ∆x and ∆y are the deviations of the x and y coordinates between two successive frames. The lengths of the vectors of the moving velocities (m/s) of the upper and lower jaws were obtained from the velocity calculated above as v = (v_x_^2 ^+ v_y_^2^)^0.5^. The gape angle, which is the angle of two lines connecting the jaw articulation and the anterior tips of the upper and lower jaws, was measured in the 2008 shark.

### Specimens examined and dissected

For further confirmation of the jaw movements and for proportional measurements, three uncatalogued and the following five catalogued specimens were used: HUMZ 197855 (Fish Collection of the Hokkaido University Museum), a 1209 mm TL female from Tokyo Bay, Japan; HUMZ 204615, a 1315 mm TL female from Tokyo Bay, Japan; HUMZ 215085, a 1355 mm TL female from Tokyo Bay, Japan; HUMZ 221262, a 1295 mm TL female from Sagami Bay, Japan; and YCM-P12237 (Fish Collection of Yokosuka City Museum), a 2710 mm TL male from Sagami Bay, Japan. All the specimens above were collected by the commercial fisheries, brought in the fish market dead, and were obtained.

## Additional Information

**How to cite this article**: Nakaya, K. *et al*. Slingshot feeding of the goblin shark *Mitsukurina owstoni* (Pisces: Lamniformes: Mitsukurinidae). *Sci. Rep*. **6**, 27786; doi: 10.1038/srep27786 (2016).

## References

[b1] Jordan D. S. Description of a species of fish (*Mitsukurina owstoni*) from Japan, the type of a distinct family lamnoid sharks. Proc. Calif. Acad. Sci. 3^rd^ ser. Zool 1, 199–201 (1898).

[b2] Last P. R. & Stevens J. D. Sharks and Rays of Australia (Harvard Univ. Press 2009).

[b3] Castro J. I. The Sharks of North Carolina. Oxford Univ. Press (2011).

[b4] Ebert D. A., Fowler S. & Compagno L. J. V. Sharks of the World (Wild Nature Press 2013).

[b5] Shirai S. & Nakaya K. Functional morphology of feeding apparatus of the cookie-cutter shark, *Isistius brasiliensis* (Elasmobranchii, Dalatiinae). Zool. Sci. 9, 811–821 (1992).

[b6] Shirai S. & Okamura O. Anatomy of *Trigonognathus kabeyai*, with comments on feeding mechanism and phylogenetic relationships (Elasmobranchii, Squalidae). Japan. J. Ichthyol. 39, 139–150 (1992).

[b7] Wu E. H. Kinematic analysis of jaw protrusion in orectolobiform sharks; a new mechanism for jaw protrusion. J. Morph. 222, 175–190 (1994). 10.1002/jmor.1052220205 29865406

[b8] Wilga C. D. Morphology and evolution of the jaw suspension in lamniform sharks. J. Morph. 265, 102–119 (2005). 15880740 10.1002/jmor.10342

[b9] Wilga C. D. Hyoid and pharyngeal arch function during ventilation and feeding in elasmobranchs: conservation and modification in function. J. Applied Ichthyol. 266, 162–166 (2010).

[b10] Nakaya K., Suda K. & Matsumoto R. Feeding strategy of the megamouth shark *Megachasma pelagios* (Lamniformes: Megachasmidae). J. Fish. Biol. 73, 17–34 (2008).

[b11] Motta P. J. & Wilga C. D. Advances in the study of feeding behaviors, mechanisms, and mechanics of sharks. Environ. Biol. Fish. 60, 131–156 (2001).

[b12] Motta P. J., Hueter R. E., Tricas T. C. & Summers A. P. Kinematic analysis of suction feeding in the nurse shark, *Ginglymostoma cirratum* (Orectolobiformes, Ginglymostomatidae). Copeia 2002, 24–38 (2002).

[b13] Wilga C. D., Wainwright P. C. & Motta P. J. Evolution of jaw depression mechanics in aquatic vertebrates: insights from Chondrichthyes. Biol. J. Linnean Soc. 71, 165–185 (2000).

[b14] Wilga C. D. A functional analysis of jaw suspension in elasmobranchs. Biol. J. Linnean Soc. 78, 483–502 (2002).

[b15] Springer V. G. & Gold J. P. Sharks in Question (Smithsonian Institution Press, 1989).

[b16] Motta P. J. In Biology of Sharks and their Relatives (eds Carrier J. C..). Prey capture behavior and feeding mechanics of elasmobranchs, 165–202 (CRC Press, 2004).

[b17] Motta P. J. & Huber D. H. In Biology of Sharks and Their Relatives 2^nd^ edn (eds Carrier J. C. .). Prey capture behavior and feeding mechanics of elasmobranchs, 153–209 (CRC Press, 2012).

[b18] Compagno L. J. V. Sharks of the world. Vol. 2. Bullhead, mackerel and carpet sharks (Heterodontiformes, Lamniformes and Orectolobiformes). FAO Species Catalog. Fish. Purp. *no.1*, 2, 1–269 (2001).

[b19] Wilga C. D. & Motta P. J. Conservation and variation in the feeding mechanism of the spiny dogfish *Squalus acanthias*. J. Exp. Biol. 201, 1345–1358 (1998). 9547315 10.1242/jeb.201.9.1345

[b20] Goto T. Comparative anatomy, phylogeny and cladistic classification of the order Orectolobiformes (Chondrichthyes, Elasmobranchii). Mem. Grad. School Fish. Sci. Hokkaido Univ. 48, 1–100 (2001).

[b21] Dean M. N. & Motta P. J. Feeding behavior and kinematics of the lesser electric ray, *Narcine brasiliensis* (Elasmobranchii: Batoidea). Zool. 107, 171–189 (2004). 10.1016/j.zool.2004.04.002 16351936

[b22] Westneat M. W. & Wainwright P. C. Feeding mechanism of *Epibulus insidiator* (Labridae; Teleostei): Evolution of a novel functional system. J. Morph. 202, 129–150 (1989). 10.1002/jmor.1052020202 29865677

[b23] Tricas T. C. & McCosker J. E. Predatory behavior of the white shark (*Carcharodon carcharias*), with notes on its biology. Proc. Calif. Acad. Sci. 43, 221–238 (1984).

[b24] Motta P. J., Tricas T. C., Hueter R. E. & Summers A. P. Feeding mechanism and functional morphology of the jaws of the lemon shark *Negaprion previrostris* (Chondrichthyes, Carcharhinidae). J. Exp. Biol. 200, 2765–2780 (1997). 9326502 10.1242/jeb.200.21.2765

[b25] Wilga C. D., Motta P. J. & Sanford C. P. Evolution and ecology of feeding in elasmobranchs. Integr. Comp. Biol. 47, 55–69 (2007). 21672820 10.1093/icb/icm029

[b26] Yano K., Miya M., Aizawa M. & Nochi T. Some aspects of the biology of the goblin shark, *Mitsukurina owstoni*, collected from the Tokyo Submarine Canyon and adjacent waters. Japan. Ichthyol. Res. 54, 388–398 (2007).

[b27] Compagno L. J. V., Dando M. & Fowler S. Sharks of the World (Princeton Univ. Press, Princeton, 2005).

[b28] Compagno L. J. V. In Interrelationships of Fishes (eds Greenwood P. H..). Interrelationships of living elasmobranchs, 15–61 (Academic Press, 1973).

[b29] Compagno L. J. V. Sharks of the Order Carcharhiniformes (Princeton Univ. Press, 1988).

[b30] Naylor G. J. P. . A DNA sequence-based approach to the identification of shark and ray species and its implications for global elasmobranch diversity and parasitology. Bull. Amer. Mus. Nat. Hist. 367, 1–262 (2012).

[b31] Naylor G. J. P. . In Biology of Sharks and Their Relatives 2nd edn (eds Carrier J. C. .). Elasmobranch phylogeny: a mitochondrial estimate based on 595 species, 31–56 (CRC Press, 2012).

[b32] Compagno L. J. V. In Elasmobranchs as Living Resources: Advances in the Biology, Ecology, Systematics, and the Status of the Fisheries (eds Pratt H. L. Jr. .). Relationships of the megamouth shark, *Megachasma pelagios* (Lamniformes: Megachasmidae), with comments on its feeding habit, 357–379 (NOAA Tech. Rep. NMFS 90, 1990).

[b33] Capetta H., Duffin C. & Zidek J. Chondrichthyes in Fossil Record 2 (ed. Benton J.), 593–609 (1993).

[b34] Kriwet J. Neoselachier (Pisces, Elasmobranchii) aus der Unterkreide (unteres Barremium) von Galve und Alcaine (Spanien, Provinz Teruel). Palaeo. Ichthyol. 9, 113–142 (1999).

[b35] Rees J. Neoselachian shark and ray teeth from the Valanginian, Lower Cretaceous, of Wawal, Central Poland. Palaeontol. 48, 209–221 (2005).

[b36] Schmitz L., Thies D. & Kriwet J. Two new lamniform sharks (*Leptostyrax stychi* sp. nov. and *Protolamna sarstedtensis* sp. nov.) from the early Cretaceous of NW Germany. *Neues Jahrbuch für* Geologie und Paläontologie–Abhandlungen 257, 283–296 (2010).

[b37] Helfman G. S., Collette B. B. & Facy D. E. The Diversity of Fishes (Blackwell Science, 2000).

